# Raman Spectroscopic Analysis of Molecular Structure and Mechanical Properties of Hypophosphatasia Primary Tooth

**DOI:** 10.3390/molecules29246049

**Published:** 2024-12-22

**Authors:** Hayata Imamura, Tetsuya Adachi, Wenliang Zhu, Toshiro Yamamoto, Narisato Kanamura, Hiroaki Onoda, Aki Nakamura-Takahashi, Masataka Kasahara, Masaru Nakada, Hideo Sato, Giuseppe Pezzotti

**Affiliations:** 1 Ceramic Physics Laboratory, Kyoto Institute of Technology, Sakyo-ku, Matsugasaki, Kyoto 606-8585, Japan; d2871002@edu.kit.ac.jp (H.I.); wlzhu@kit.ac.jp (W.Z.); 2 Department of Dental Medicine, Graduate School of Medical Science, Kyoto Prefectural University of Medicine, Kamigyo-ku, Kyoto 602-8566, Japan; yamamoto@koto.kpu-m.ac.jp (T.Y.); kanamura@koto.kpu-m.ac.jp (N.K.); pezzotti@kit.ac.jp (G.P.); 3 Department of Dentistry, Kyoto Prefectural Rehabilitation Hospital for Mentally and Physically Disabled, Naka Ashihara, Joyo 610-0113, Japan; 4 Department of Immunology, Graduate School of Medical Science, Kyoto Prefectural University of Medicine, Kamigyo-ku, 465 Kajii-cho, Kyoto 602-8566, Japan; 5 Department of Biomolecular Chemistry, Faculty of Science and Technology, Kyoto Prefectural University, 1-5, Shimogamo-nakaragi, Sakyo-ku, Kyoto 606-8522, Japan; h-onoda@kpu.ac.jp; 6 Department of Pharmacology, Tokyo Dental College, 2-9-18 Kandamisaki-cho, Chiyoda-ku, Tokyo 101-0061, Japan; atakahashi@tdc.ac.jp (A.N.-T.); mkasahar@tdc.ac.jp (M.K.); 7 Toray Research Center, Inc., 2-11 Sonoyama 3-chome, Otsu 520-8567, Japan; masaru.nakada.n3@trc.toray; 8 Department of Pediatric Dentistry, Kagoshima University Hospital, 8-35-1 Sakuragaoka, Kagoshima 890-8544, Japan; hideo-sato@dent.kagoshima-u.ac.jp; 9 Biomedical Engineering Center, Kansai Medical University, 1-9-11 Shin-machi, Hirakata, Osaka 573-1191, Japan; 10 Department of Orthopedic Surgery, Tokyo Medical University, 6-7-1 Nishi-Shinjuku, Shinjuku-ku, Tokyo 160-0023, Japan; 11 Department of Applied Science and Technology, Politecnico di Torino, Corso Duca degli Abruzzi 24, 10129 Torino, Italy; 12 Department of Molecular Science and Nanosystems, Ca’ Foscari University of Venice, Via Torino 155, 30172 Venice, Italy

**Keywords:** collagen, hydroxyapatite, hypophosphatasia, Raman spectroscopy

## Abstract

Mild hypophosphatasia (HPP) can be difficult to distinguish from other bone disorders in the absence of typical symptoms such as the premature loss of primary teeth. Therefore, this study aimed to analyze the crystallinity of hydroxyapatite (HAp) and the three-dimensional structure of collagen in HPP teeth at the molecular level and to search for new biomarkers of HPP. Raman spectroscopy was used to investigate the molecular structure, composition, and mechanical properties of primary teeth from healthy individuals and patients with HPP. The results showed that the crystallinity of HAp decreased and the carbonate apatite content increased in the region near the dentin–enamel junction (DEJ) of HPP primary teeth. X-ray diffraction (XRD) analyses confirmed a decrease in HAp crystallinity near the DEJ, and micro-computed tomography (CT) scanning revealed a decrease in mineral density in this region. These results suggest incomplete calcification in HPP primary dentin and may contribute to the development of diagnostic and therapeutic agents.

## 1. Introduction

Hypophosphatasia (HPP) is a type of bone system disease in which low bone calcification occurs throughout the body due to the decreased activity of tissue-nonspecific alkaline phosphatase (TNSALP), an enzyme that promotes bone calcification [1,2,3,4,5]. TNSALP is a key enzyme in skeletal tissue calcification and is involved in the dephosphorylation of various physiological substrates. It has important physiological functions, including extra-skeletal functions such as neuronal development, lipopolysaccharide detoxification, anti-inflammatory effects, bile pH regulation, and blood–brain barrier maintenance. It is also involved in the ectopic pathological calcification of soft tissues, especially the vasculature [6]. In healthy individuals, TNSALP breaks down the body’s inorganic pyrophosphate into phosphate, which binds to calcium, resulting in calcification and bone formation [7,8,9,10]. In HPP, however, inorganic pyrophosphate is not broken down and cannot combine with calcium, so calcification does not occur normally and bone formation is incomplete [11,12,13,14,15]. Deficiency in TNSALP can cause seizures and other symptoms in HPP patients, and in severe cases, death may result from intractable bone fractures or respiratory distress due to defective rib formation [16,17,18,19,20]. The most characteristic symptom of HPP is the premature loss of primary teeth [21,22,23,24]. In general, primary teeth are lost at the age of 5–6 years old due to root resorption, but in the case of HPP, the roots are not resorbed and are lost at the age of 4 or younger [25,26,27,28,29], as the premature loss of primary teeth occurs painlessly, bloodlessly, and with the root intact because deficient mineralization of the cementum impairs their linkage to the periodontal ligament.

To date, most conventional studies on HPP have focused on enzymes and genetic mutations, while the variation in and distribution of tooth structure have been investigated insufficiently. In addition, the alkaline phosphatase (ALP) enzyme replacement therapy asfotase alfa (STRENSIQ^®^, Alexion Pharmaceuticals Inc., Boston, MA, USA) has now been established, and it is now possible to save the lives of severely ill patients who were previously difficult to save [30,31,32,33,34]. ALXN1850, a next-generation ALP enzyme replacement therapy with greater activity against substrates than asfotase alfa, is currently being tested in clinical trials worldwide [35]. However, enzyme replacement therapy is not a curative therapy because it produces antibodies in the body that gradually diminish the effectiveness of the therapy. It also imposes a heavy financial and physical burden on patients [36,37,38,39].

Furthermore, HPP is often misdiagnosed, partly because its symptoms are similar to those of osteoporosis and because HPP is not well known. However, the osteoporosis drug bisphosphonate is known to be counterproductive when used for HPP. For example, Ng et al. have reported that 3% of clinic patients with osteoporosis and with low serum ALP were diagnosed with hypophosphatasia. Low ALP can be exacerbated by anti-osteoporotic therapy, and the recognition of low ALP can help to avoid misdiagnosis and incorrect treatment [40]. As HPP is a hereditary disease, and there are many individuals with potential genetic mutations, it is imperative to establish a simple diagnostic technique [41,42].

Therefore, we proposed using Raman spectroscopy to clarify the variations in the chemical composition and structure of HPP teeth. Raman spectroscopy is a non-destructive method used to analyze the molecular structure of materials and is suitable for an analysis of the structure of hydroxyapatite and collagen [43,44]. Normally, the crystallinity of HAp and the steric orientation of collagen cannot be studied by staining or genetic testing, but Raman spectroscopy is sensitive to these factors and can be analyzed, as previous research suggested that it is effective in detecting caries and demineralization. In this study, we analyzed the structure of hydroxyapatite or collagen and the mineral density of HPP patients’ primary teeth.

## 2. Results

### 2.1. Micro-Computed Tomography (CT) Analyses

**Figure 1:**
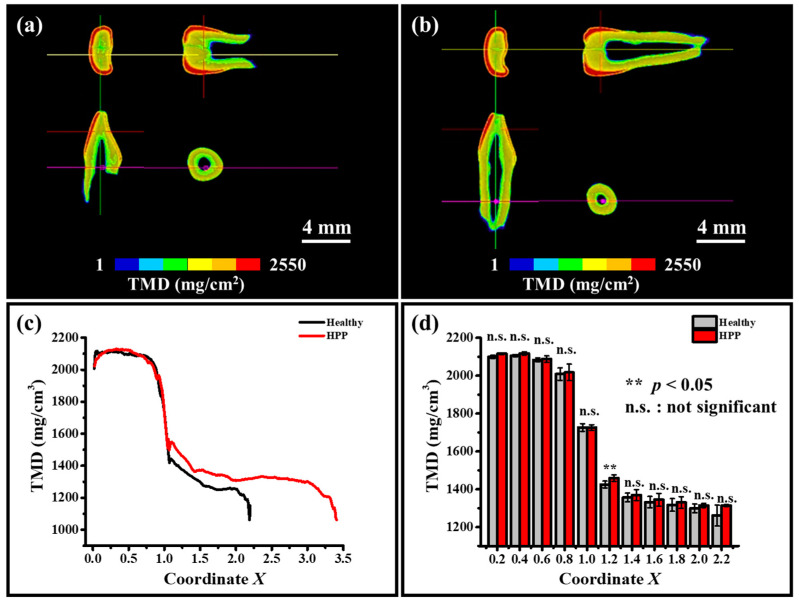
Three-dimensional color maps of micro-CT scans of (**a**) healthy primary teeth and (**b**) HPP primary teeth. (**c**) Plots of TMD with coordinate *X*. (**d**) Comparison of TMD variations between healthy and HPP primary teeth (n.s.—not significant).

Micro-CT images were taken to check for caries and unnatural destruction. Color maps of the micro-CT scans, shown in Figure 1a,b, confirmed that there was no destruction of tooth shape, such as by enamel demineralization or caries. Moreover, from these results, the value of the enamel length *L* could be determined. The results for tissue mineral density (TMD) plotted against the *X* coordinate are shown in Figure 1c, and a comparison of the average TMD between healthy and HPP primary teeth is shown in Figure 1d. The color maps and the fact that TMD was not detected at *X* > 2.8 in healthy primary teeth confirmed that root resorption had occurred in healthy primary teeth. TMD was significantly higher for HPP teeth at *X* = 1.2 in the dentin region.

Data on healthy dentin were only available up to *X* = 2.2–2.6, because of the loss of the roots. As can be seen in Figure 1c,d, TMD remained almost constant in the enamel region, while it rapidly and markedly decreased across the DEJ and tended to be insignificantly varied in dentin. In addition, compared to healthy teeth, HPP samples showed a relatively higher TMD at around *X* = 1.2. Raman spectroscopic line scan was used to further analyze the molecular structural variation in the tooth root induced by HPP.

### 2.2. Synchrotron Radiation Nano-CT Analyses

**Figure 2:**
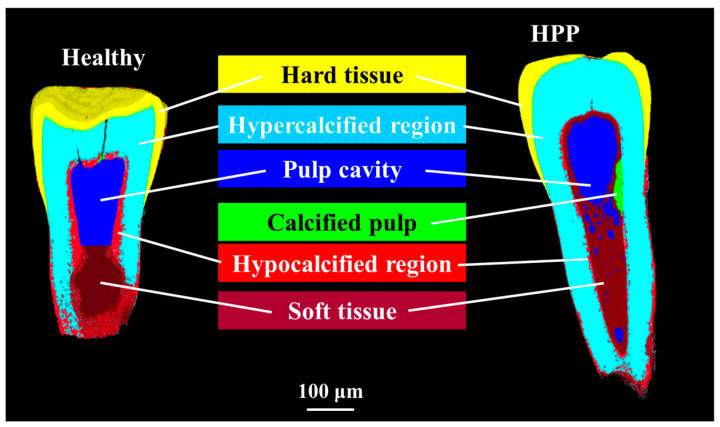
Nano-CT images and 3D color maps of healthy and HPP primary teeth.

Nano-CT images and 3D color maps of the investigated incisors are shown in Figure 2. In the HPP sample, the relative volume of calcified tissue and soft tissue in the pulp was significantly higher than that in the healthy one.

Although the TMD of HPP teeth was higher than that of the healthy group (*p* < 0.05), the crystallinity was lower than that of the healthy group (*p* < 0.05).

### 2.3. X-Ray Diffraction (XRD) Analyses

**Figure 3:**
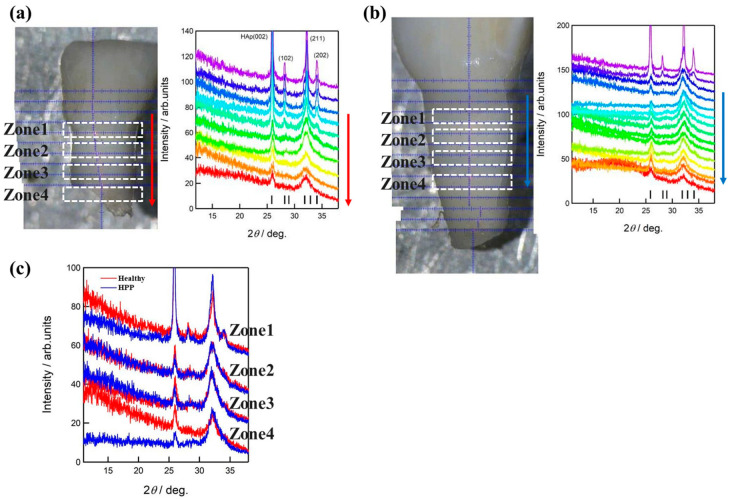
XRD patterns at different locations of (**a**) healthy primary teeth and (**b**) HPP primary teeth. The patterns approach the root according to the direction of the arrows. (**c**) Comparison of the patterns in different regions shown by the dashed lines.

The XRD patterns of the samples are shown in Figure 3. Similarly to the results of Raman analysis, the diffraction of low-crystalline HAp was observed in the root and that of high-crystalline HAp in the crown. Diffraction from the (002) plane was strongly observed in the crown, suggesting that the *c*-axis of HAp was oriented normally to the surface. The (002) diffraction of HPP was smaller than the (211) diffraction at the root of the tooth, suggesting that the *c*-axis orientation was low or that the (002) plane was not well formed. Additionally, 2θ = 15–25° broad scattering was observed at two spectra near the bottom of the HPP root, suggesting the presence of an amorphous structure.

### 2.4. Raman Spectroscopic Analyses

**Figure 4:**
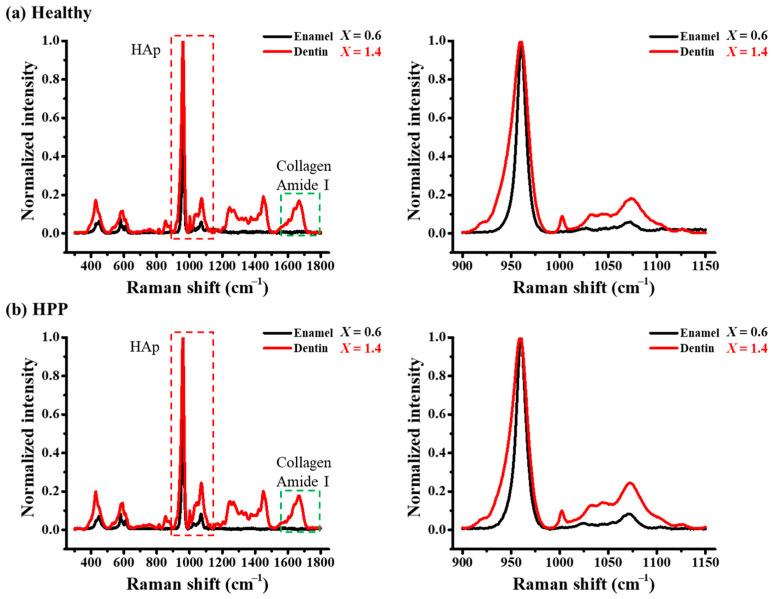
Typical Raman spectra collected at the enamel and dentin of (**a**) healthy primary teeth and (**b**) HPP primary teeth. The red and green dashed lines enclose bands indicating apatite and collagen, respectively.

**Figure 5:**
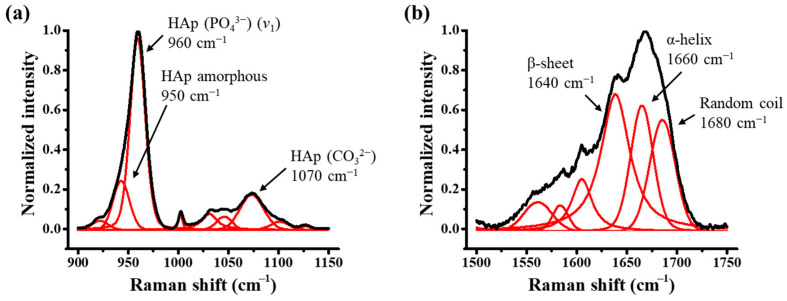
Spectral deconvolution of Raman spectra related to (**a**) apatite and (**b**) collagen at *X* = 1.4.

**Figure 6:**
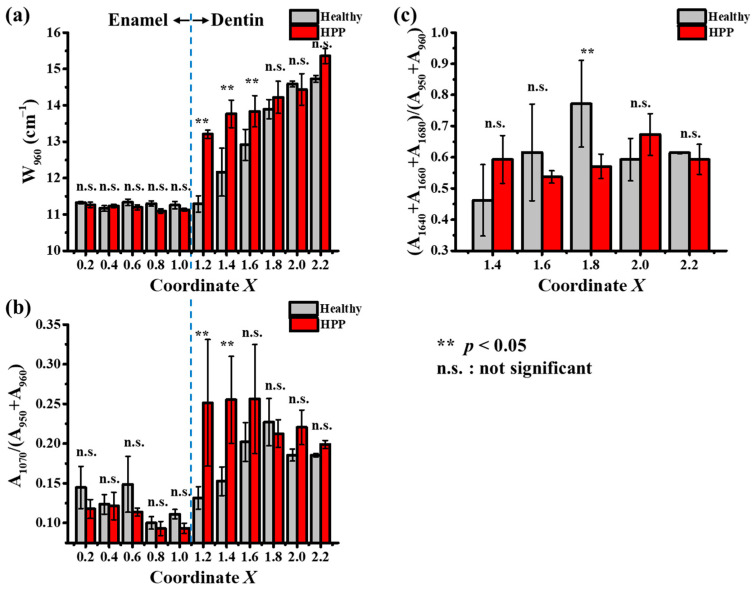
Raman data plots of (**a**) W_960_, (**b**) carbonate to phosphate ratio, and (**c**) collagen to apatite ratio for healthy and HPP primary teeth. The blue dashed line shows the dentin–enamel junction.

Typical Raman spectra of enamel and dentin for healthy and HPP teeth are shown in Figure 4. In the enamel region, Raman peaks were observed in the range < 1100 cm^−1^, which indicated the vibrations of PO_4_ in HAp, while in dentin, several additional bands induced by collagen appeared at higher wavenumbers. Figure 5a shows a representative spectral deconvolution of the *v*_1_ mode originating from a P–O stretching vibration, which includes a strong band at around 960 cm^−1^, indicating the crystalline phase of HAp, and a sub-band at around 950 cm^−1^, indicating the amorphous phase of HAp [43,44]. In dentin, the bands of Amide III (1200–1300 cm^−1^), Amide II (1400–1500 cm^−1^), and Amide I (1600–1700 cm^−1^) were all attributed to collagen proteins. Amide I exhibits several sub-bands attributed to a β-sheet (1640 cm^−1^), an α-helix (1660 cm^−1^), and a random coil (1680 cm^−1^) (cf. Figure 6b). No collagen bands were identified as the enamel was composed of approximately 96% HAp. The full width at half maximum (FWHM) of 960 cm^−1^ (W_960_) was used to indicate HAp crystallinity, because smaller values of W_960_ indicate higher crystallinity [43,44,45]. Furthermore, as a band indicative of carbonate apatite appeared at 1070 cm^−1^, the carbonate apatite content could be evaluated using A_1070_/(A_950_ + A_960_), that is, the ratio of the carbonate-related band area to the sum of the band areas of the amorphous and crystalline phases of HAp [45]. The collagen content relative to apatite was evaluated by (A_1640_ + A_1660_ + A_1680_)/(A_950_ + A_960_). These Raman data plots against the coordinate *X* are shown in Figure 6. W_960_ showed a pronounced increase in dentin (i.e., *X* > 1), especially at *X* = 1.2, 1.4, and 1.6 for HPP teeth, indicating a significantly lower crystallinity of HAp (Figure 6a). In addition, the carbonate apatite content also increased significantly in these areas on HPP teeth with respect to the healthy cases (Figure 6b). Therefore, this suggests that HPP may result in a decrease in HAp crystallinity and an increase in carbonate apatite in the area close to the dentin–enamel junction (DEJ) of dentin. Concerning the variation of collagen content, the areal ratio of (A_1640_ + A_1660_ + A_1680_)/(A_950_ + A_960_) showed insignificant variation (cf. Figure 6c), indicating a negligible variation in dentin collagen induced by HPP.

### 2.5. Indentation Test

**Figure 7:**
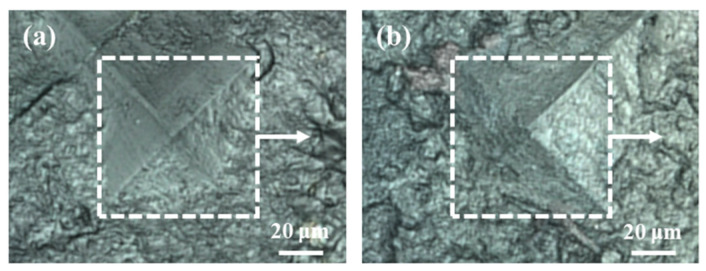
Microscopic images around Vickers indentation on (**a**) healthy primary teeth and (**b**) HPP primary teeth. The arrows and dashed squares show the orientation of the Raman scan and indentation zone, respectively.

To examine the influence of HPP on mechanical properties and microstructure in this area of dentin near the DEJ, Vickers indentations were introduced on the surfaces of the investigated healthy and HPP teeth. Microscopic images of the Vickers indentations are shown in Figure 7, exhibiting similar square-like profiles and no crack generation around the indentation.

**Figure 8:**
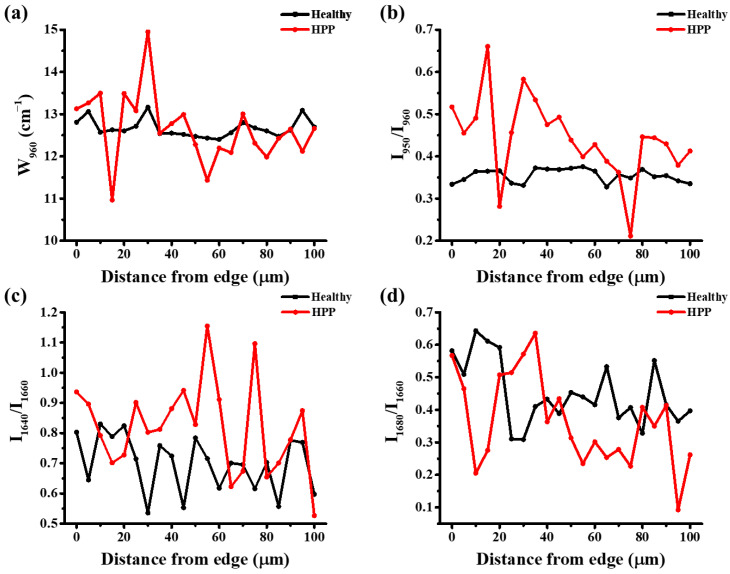
Variations in (**a**) W_960_, (**b**) I_950_/I_960_, (**c**) I_1640_/I_1660_, and (**d**) I_1680_/I_1660_ in the line scans with distance from the indentation edge.

Accordingly, spectral line scans were conducted crossing the indentation edge, avoiding the indentation. As shown in Figure 8, the FWHM of W_960_ and the ratio of I_950_/I_960_ showed much greater scattering with distance from the indentation edge in HPP primary teeth than in healthy primary teeth. In addition, the scattering in HPP primary teeth was also larger than that in the same region without the indentation, but no significant variations could be found in the case of healthy teeth (cf. Figure 6a and Figure 8a). Furthermore, HPP teeth showed much higher values of I_950_/I_960_ near the indentation corner, and the ratio decreased with distance from the edge, indicating a heavier lattice distortion induced by indentation in HPP teeth. Note that because the tooth surfaces of the investigated samples were not sufficiently flat, the indentations are somewhat asymmetrical. Accordingly, the variation of stress field may have caused somewhat different shifts in the position of the HAp v_1_ band at 960 cm^−1^, but the width of the band and the relative intensity were insignificantly influenced.

Concerning the impact on collagen, as seen in Figure 8c,d, the ratios of I_1640_/I_1660_ and I_1680_/I_1660_ showed minor variations with distance from the indentation edge in HPP and healthy teeth.

**Figure 9:**
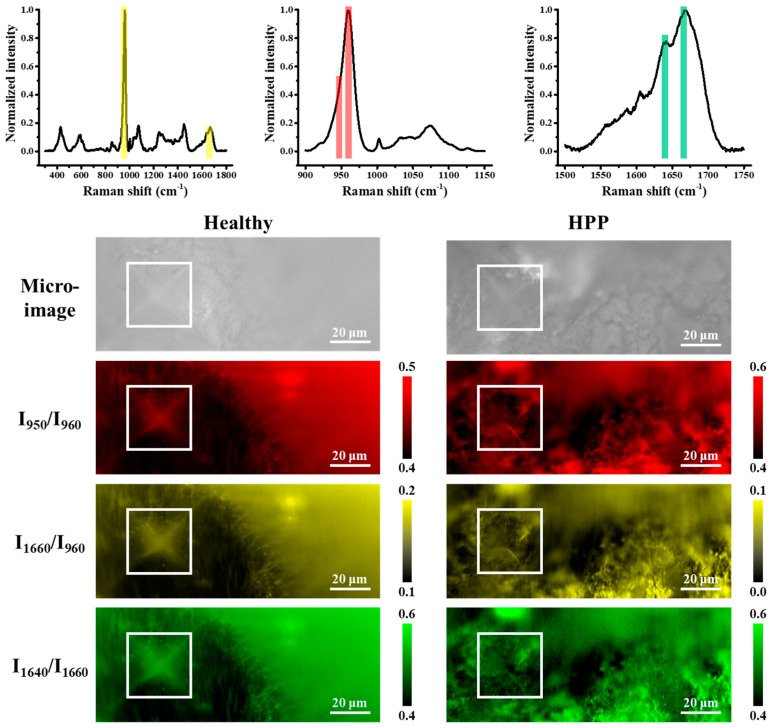
Microscope imaging and Raman imaging of different intensity ratios around the indentation generated for HPP and healthy teeth. The white squares show indentation zones. The color bars in the spectra correspond to the colors of Raman images.

To further examine the reliability of the observed variations, Raman imaging was carried out around the investigated indentation. Figure 9 shows the distribution of band intensity ratios (I_950_/I_960_: amorphous/crystal, I_1660_/I_960_: collagen/apatite, I_1640_/I_1660_: β-sheet/α-helix) around the indentation. Raman imaging showed that stress relaxation was observed in healthy primary teeth, but not in HPP primary teeth. This is because the relative intensity was almost constant at distances greater than 20 μm from the edge of the indentation in healthy primary teeth.

## 3. Discussion

Genetic diagnosis and biological examination on serum ALP levels have been performed previously for HPP. For example, Okawa et al. reported a method to easily evaluate alveolar bone mineralization in HPP patients using orthopantomography and HAp phantom, imaging tests commonly performed in dentistry. In this method, orthopantomographic images of 200 healthy subjects (aged 2–15 years) were collected, and the diagnosis of HPP patients was based on their pixel values [22]. However, this method is limited to identification by the degree of calcification in specific areas on the panoramic X-ray, and it failed to analyze the crystallinity of HAp and the steric orientation of collagen, so it was difficult to distinguish osteoporosis and other bone system diseases. Therefore, we proposed a new method of identification based on differences in crystal structure (an evaluation of crystallinity) using Raman spectroscopic analysis, which may be applicable as a biomarker in the diagnosis of HPP in vitro.

Raman spectroscopic analyses revealed a reduced crystallinity and an increased amorphization of HAp in dentin from the DEJ to the root, confirmed by the XRD results (cf. Figure 3 and Figure 6). In addition, the amorphization of HAp in the dentin around the DEJ showed a similar variation in its trend to that of carbonation in HPP teeth. As can be seen in Figure 6a, along with the increase in the *v*_1_ bandwidth, W_960_, HAp crystallinity decreased from the DEJ to the root in both healthy and HPP teeth. Moreover, HAp crystallinity was significantly lower in HPP teeth at *X* = 1.2, 1.4, and 1.6 in dentin, while carbonate apatite contents were increased significantly in HPP teeth at these sites. That is to say, HPP resulted in a decrease in crystallinity and an increase in carbonate apatite in the region close to the DEJ of dentin. Indeed, similar phenomena could be observed in HPP mice: we analyzed osteoblasts collected from healthy and HPP mice with large differences in femur bone density (cf. CT and Raman images in Supplementary Materials Figure S2), and Raman analysis also revealed that HPP subjects had less HAp crystallinity than healthy subjects, confirming the applicability of apatite crystallinity and carbonate apatite as a new biomarker in HPP diagnosis. This might be related to the incomplete calcification and bone formation in HPP, which results in the occurrence of carbonation. Indeed, Rolvien et al. reported that carbonate apatite content was increased in HPP teeth [5]. However, the present results revealed that such variations significantly differ among different locations on the teeth. In normal biomineralization, calcification progresses as hydroxyapatite, formed by the combination of phosphate and calcium, is deposited on collagen fibers in dentin and grows. The increase in the ratio of carbonate apatite may be due to a decrease in ALP. It is believed that a lack of phosphate inhibits normal biomineralization and causes it to be replaced by carbonate. In the region of *X* > 1.8, a simple comparison with HPP primary teeth is difficult because of the severe root resorption and the significant decrease in the crystallinity of healthy primary teeth.

Conversely, no significant variation induced by HPP was observed for collagen content associated with the amorphization of HAp in the dentin around the DEJ because the carbonation process took place in HAp. Indeed, in the region of 1.2 < *X* < 1.6, with respect to healthy primary teeth, when protein variations in different regions of HPP teeth were analyzed, the collagen/HAp areal ratio did not vary significantly with HAp amorphization (cf. Figure 6c). The band intensity of HAp decreases with HAp amorphization, and the protein content is expected to decrease accordingly.

Pezzotti et al. reported that indentation in osteoporotic bones makes stress relaxation more difficult than in healthy bones [43]. The present results showed that HPP primary teeth produced a larger shift in Raman peaks for HAp than healthy teeth. The larger peak shift for HPP is thought to be due to the generation of higher residual stress by the applied load. Raman imaging shows a difference in Raman profiles between the area around the indentation and the area away from the indentation in the healthy case, but not in the HPP case (cf. Figure 9). Note that for Vickers indentation, the presence of four corners can reveal the distribution of stress upon loading and stress relaxation along different directions. The observed similar profiles around the indentation in different directions, and the above-observed difference concerning locations far away, suggest that HPP is considered as difficult for stress relaxation as osteoporosis.

Note that such observed differences might also be associated with the variation in the TMD of the tooth. It is generally believed that mineral density decreases in the presence of bone system diseases. However, as demonstrated in Figure 1, a micro-CT analysis of teeth showed that TMD was higher in HPP teeth compared to in healthy ones, possibly caused by the internal resorption of the tooth root in healthy teeth and the formation/mineralization of calcified tissue in some part of the pulp in HPP teeth (cf. Figure 2), according to the results of further nano-CT imaging using synchrotron radiation.

In practice, teeth are fixed to the jawbone via the periodontal ligament. Cementum, which covers the outer surface of the tooth root, plays a role in attaching the periodontal ligament to the tooth root. The prevailing theory is that in HPP, cementum is hypoplastic, which impairs the adhesion between the tooth and the jawbone, and it is thought that the teeth become loose and fall out [23]. In addition, although not as frequently as primary teeth, permanent teeth may also fall out. However, the results of this study did not allow us to consider the influence of cementum. In contrast to the prevailing theory, the finding that cementum dysplasia does not occur, but rather that cellular cementum strays into the central portion of the root, has recently been reported [46]. EVs released from ectopic cellular cementum may inhibit osteoclasts or odontoclasts, and thereby inhibit root resorption and internal resorption [47,48]. It is becoming increasingly difficult to obtain healthy, unabsorbed primary teeth. In the future, it will be necessary to spectroscopically analyze cementum through histological analysis using an HPP model (TNSALP knockout mice) [21].

Osteopontin (OPN) is a mineralization inhibitor that increases with decreased TNSALP activity. Phosphorylated osteopontin (p-OPN) binds directly to hydroxyapatite and prevents bone mineralization [49]. It is present in kidney stones and is involved in their formation [50]. TNSALP dephosphorylates p-OPN and converts it to OPN. Therefore, the decrease in HAp crystallinity and increase in carbonate apatite in HPP with decreased TNSALP activity may be explained by increased OPN in bone tissue [51]. Raman spectra of OPN were obtained, showing two sharp bands in the spectral range from 800 to 900 cm^−1^. However, when these were compared to those of primary teeth, no significant differences were found between the spectra of healthy and HPP primary teeth, possibly due to a low amount of OPN.

Finally, it is worth noting that primary teeth can be easily and non-invasively collected, so there are many opportunities to collect them, and strict management is not required for spectroscopic analysis. All teeth used in this study were primary incisors, but the difference in the DEJ between molars and incisors may cause a somewhat different population of apatite crystallinity, which needs further confirmation. In addition, given that a fiber Raman probe is available along with the rapid development of portable Raman spectroscopes nowadays, in situ Raman analysis of HPP may become feasible because the spectral deconvolution of the obtained Raman spectra collected at different locations enables the quantitative analysis of both the bandwidth and the relative intensity. This allows it to be applied in the screening of potential HPP patients. Therefore, Raman spectroscopy could be promising for the more convenient and rapid diagnosis of HPP in the future.

## 4. Experimental Procedures

### 4.1. Samples

**Figure 10:**
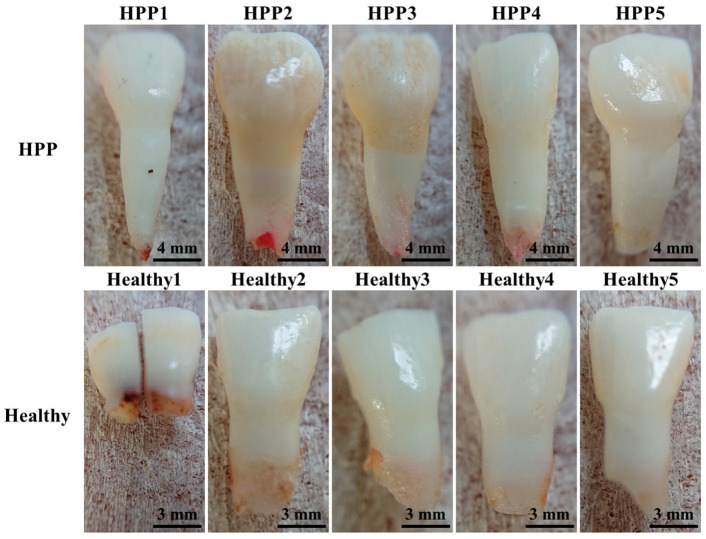
Optical images of the investigated healthy and HPP primary teeth. Healthy1 was damaged after micro-CT imaging, but other results were not affected.

This study was conducted in accordance with the Declaration of Helsinki and approved by the Ethics Committee of the Kyoto Prefectural University of Medicine (protocol code: ERB-C-136; and date of approval: 14 March 2014) and the Kyoto Prefectural Rehabilitation Hospital For Mentally and Physically Disabled (protocol code: RBMR17 and date of approval:2020.1.10). Informed consent was obtained from all subjects involved in the study. In addition, written informed consent was obtained from all patients to publish this paper. In this study, five primary teeth (all incisors) from HPP patients and five from healthy donors were used as the test teeth. Pathological primary teeth of patients diagnosed with HPP were collected at Kagoshima University Hospital. Patients diagnosed with HPP did not have any overlapping systemic diseases or syndromes other than the diagnosis of HPP. All sample patients (subjects) had no prior experience with high-dose antibiotics or other medications. In addition, Japanese tap water does not contain fluoride, and they had only experienced exposure to food, which is a natural source of fluoride. They had no history of other medications or lifestyle habits that may have affected the hard tissues of the teeth. Because HPP is rare disease, it was extremely difficult to collect a larger sample size. The healthy teeth showed physiological root resorption and thus exhibited relatively shorter tooth lengths in comparison to the HPP teeth. The general condition of the primary teeth was unknown at the time of collection, as they had spontaneously fallen out. In addition, since HPP is a hereditary disease, the parents and their medical histories were investigated; however, it is unclear whether factors extending to the fetal period, such as antibiotic (e.g., tetracycline) intake and fluoride intake, also had an impact. Micro-CT confirmed the absence of caries and a negligible influence on the obtained results (Figure 10).

### 4.2. Micro-CT Scans

X-ray micro-CT images of the tooth samples were taken to confirm root morphology and to measure mineral density from the crown to the root using the bone mineral phantom. The Microfocus X-ray CT System (inspeXio SMX-225CT, Shimadzu Corporation, Kyoto, Japan) was used for X-ray micro-CT measurements. Additionally, 3D bone densitometry software (TRI/3D-BON-FCSCL-BMD, RATOC System Engineering, Tokyo, Japan) was used to display mineral density in 3D with color mapping and to compare mineral density distribution between HPP primary teeth and healthy primary teeth.

### 4.3. Synchrotron Radiation Nano-CT Scans

Synchrotron radiation CT analysis was performed using BL28B2 at SPring-8 (Sayo-cho, Hyogo, Japan). CT images of the samples were taken, and mineral density was displayed in 3D color maps using 3D bone densitometry software (TRI/3D-BON-FCSCL-BMD, RATOC System Engineering, Tokyo, Japan) to compare the mineral density distribution between HPP primary teeth and healthy primary teeth.

### 4.4. X-Ray Diffraction (XRD)

XRD measurements were performed using a D8 DISCOVER diffractometer and a VANTEC-500 2D solid-state detector from Bruker AXS Inc. (Billerica, MA, USA). The X-ray wavelength was 0.15418 nm, with Cu *K*α radiation, and the sample-to-detector distance was approximately 170 mm. The 2D detector was positioned at 2θ–25° and reflected X-rays were integrated for 300 s at each point. One healthy primary tooth and one HPP primary tooth were measured. The covered range was 2θ = 10–40°.

### 4.5. Raman Spectroscopy

**Figure 11:**
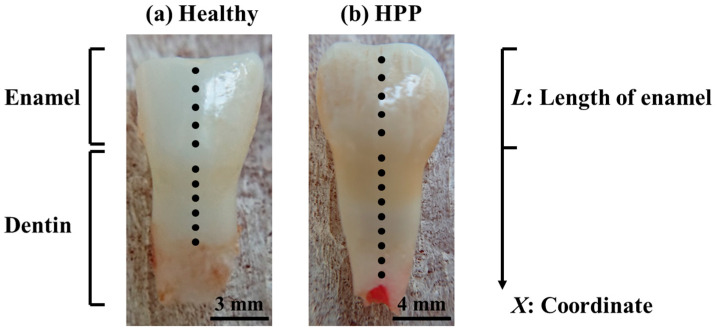
Typical Raman measurement points on (**a**) healthy primary teeth and (**b**) HPP primary teeth. The black dots show measurement points.

In this study, Raman spectra were acquired on a line from the enamel of the crown to the “dentin” (covered with cementum) of the root of the test teeth. Figure 11 shows a typical image of the acquisition points for the collection of Raman spectra of healthy and HPP teeth. The apex of the crown was set as the origin, and the distance from the origin was *x*. Accordingly, to minimize the influence of the difference in tooth length, a new coordinate was specified by *X* = *x*/*L* (*L*: the length of the enamel), because the ratio of enamel length to entire tooth length could be taken to be constant. The Raman spectra of enamel were acquired from five points at coordinates *X* = 0.2, 0.4, 0.6, 0.8, and 1.0. In addition, the Raman spectra of dentin were obtained from six points (*X* = 1.2, 1.4, 1.6, 1.8, 2.0, and 2.2). A high-resolution laser Raman microscope (T-64000, Horiba/Jobin-Yvon, Kyoto, Japan) equipped with a 1024 × 256 pixels charge-coupled device (CCD) camera (CCD-3500V, Horiba Ltd., Kyoto, Japan) and a laser with an excitation wavelength of 488 nm (Sapphire SF NX CDRH Controller, Coherent, Inc., Saxonburg, PA, USA) was used for the Raman measurements. To acquire Raman spectra, the laser output was set to 60 mW, with a pinhole aperture of 100 μm and an objective lens of ×100 magnification. The laser exposure time for the collection of a spectrum was 30 s with a totalization frequency of three times. The obtained Raman spectra were subjected to baseline subtraction and automatically deconvoluted into Gaussian–Lorentzian sub-bands. The baseline subtraction and the deconvolution procedure were performed according to the same criteria for all spectra collected on all teeth, using options available in commercial software (LabSpec 5, Horiba/Jobin-Yvon, Kyoto, Japan).

### 4.6. Indentation Test

The micro-Vickers testing machine (HMV-G, Shimadzu Corporation, Kyoto, Japan) was used to form indentations on the surface near the DEJ of the tooth samples. The test was performed by applying a load of 3 N for 10 s, followed by rapid unloading. A confocal laser microscope (VK-X100, Keyence, Osaka, Japan) was used to microscopically observe the Vickers indentation.

Then, line scans were performed at 5 µm intervals vertically, from the indentation edge to 100 µm away from the indentation, and Raman spectra were acquired. Raman imaging around the Vickers indentation was performed using a Raman microscope (RAMANtouch, Nanophoton, Osaka, Japan) to visualize the band intensity distribution associated with structural variations around the indentations.

### 4.7. Statistics

Significant difference *t*-tests on the data set were performed using the Origin software platform (Origin 2016, OriginLab Co., Northampton, MA, USA) at a significance level of *p* < 0.05.

## 5. Conclusions

In this study, the structure, composition, and mechanical properties of different primary teeth retrieved from healthy individuals and patients with hypophosphatasia were analyzed by Raman spectroscopy, XRD, and CT scans to clarify the variations in the crystallinity of HAp and the three-dimensional structure of collagen induced by HPP teeth at the molecular level and to search for new biomarkers of HPP.

The following differences were identified near the DEJ of dentin, i.e., in the area without root resorption:

1. HPP primary teeth had lower HAp crystallinity in the dentin region near the DEJ than healthy ones, even though the crystallinity decreased from the DEJ to the tooth root.
2. HPP primary teeth had higher carbonate apatite contents in the same region than healthy ones, possibly related to the incomplete calcification and bone formation in HPP and responsible for the above-mentioned decrease in HAp crystallinity.
3. HPP primary teeth had a higher TMD than healthy ones, despite an insignificant variation in collagen induced by HPP associated with the amorphization of HAp in the dentin around the junction.

It is thus believed that Raman spectroscopy will enable the more convenient and rapid diagnosis of HPP in the future and lead to the identification of potential HPP patients, as well as contribute to the development of diagnostic and therapeutic agents.

## Data Availability

The data that support the findings of this study are available on request from the corresponding authors.

## Supplementary Materials

The following supporting information can be downloaded at: https://www.mdpi.com/article/10.3390/molecules29246049/s1, Figure S1. Raman spectra of human recombinant osteopontin (OPN; FUJIFILM Wako Pure Chemical Corporation, Osaka, Japan) were obtained, showing two sharp bands in the spectral range from 800 to 900 cm^−1^. However, compared with those of primary teeth, no significant differences were found between the spectra of healthy and hypophosphatasia (HPP) primary teeth, possibly due to a low amount of OPN; Figure S2. HPP model mice were examined using Raman and micro-CT analyses; Figure S3. Fluorescence microscopic images of MSCs stained with osteopontin (green), osteocalcin (red) and Hoechst 33342 for the nuclei (blue). References [52,53,54] have been cited in the Supplementary Materials.
