# Supplementary material for: Raman Spectroscopic Analysis of Molecular Structure and Mechanical Properties of Hypophosphatasia Primary Tooth
Source: Molecules. 2024 Dec 22;29(24):6049. doi: 10.3390/molecules29246049 (PMC11678008; doi:10.3390/molecules29246049)
Supplement: Supplementary file 1 [file molecules-29-06049-s001.zip › molecules-3271747-supplementary.pdf]

Supplementary Information

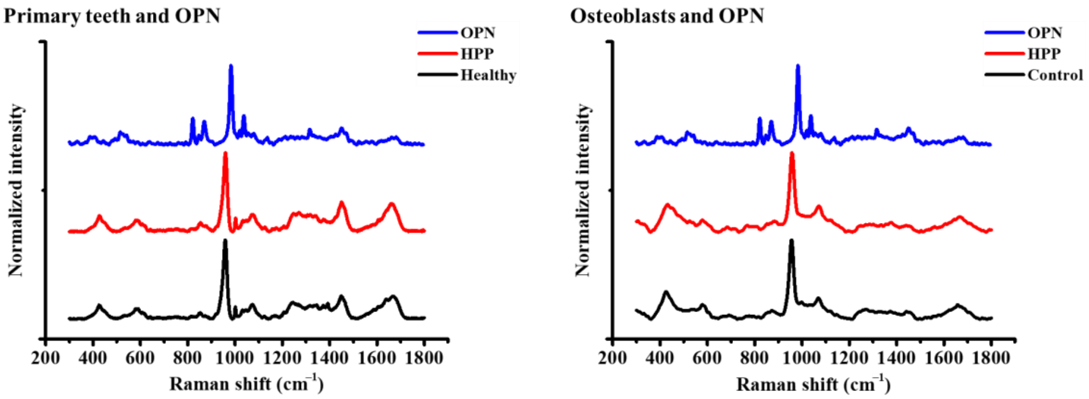

Figure S1. Raman spectra of human recombinant osteopontin (OPN; FUJIFILM Wako Pure Chemical Corporation, Osaka, Japan) were obtained, showing two sharp bands in the spectral range from 800 to 900 cm^{-1}. However, compared with those of primary teeth, no significant differences were found between the spectra of healthy and hypophosphatasia (HPP) primary teeth, possibly due to a low amount of OPN.

We used genetically engineered mice to add an insertion of a mutation in exon 6 of the alkaline phosphatase (ALP) gene. Mice were developed at the Millán laboratory [S1]. *Akp2*$^{-/-}$ mice and control *Akp2*$^{+/+}$ mice were obtained by crossing *Akp2*$^{+/-}$ mice together and genotyping by conventional methods immediately after birth [S2, S3]. These mice were bred at the Tokyo Dental College and approved by their Animal Research Ethics Committee (No. 240704). The femurs from 20-day-old *Akp2*$^{+/+}$ or *Akp2*$^{-/-}$ mice (control or HPP, respectively) were harvested, and bone density and morphology were observed using two types of micro-CT, nano3DX (Rigaku, Akishima, Japan) and inspeXio SMX-225CT (Shimadzu Corporation, Kyoto, Japan). In addition, bone marrow derived mesenchymal stromal cells (MSCs) were harvested from mouse bone marrow, seeded onto glass-bottom 6-well plates (MatTek Life Sciences, Ashland, MA, USA), and cultured in an osteogenic differentiation medium (COSMO BIO COMPANY, LIMITED, Koto-ku, Tokyo, Japan) for 4 weeks. To avoid autofluorescence from polystyrene cell culture plates, MSCs were cultured on glass substrates for Raman measurement. MSCs that had differentiated into osteoblasts were evaluated for calcification status using Raman microscopy.

Micro-CT analysis showed that the femurs of the HPP mice were shorter, less dense, and had a thin and discontinuous periosteum. Raman analysis revealed that calcified bone marrow cells were less crystalline than in the control mice. Raman imaging showed no co-localization of HAp and protein in HPP.

After 4 weeks, the osteoblasts were fixed in 4% paraformaldehyde (PFA) for 10 min at room temperature (RT). The cells were washed in phosphate-buffered saline (PBS) three times at RT and stained with anti-mouse osteocalcin (OC) Clone R21C-01A (rat) antibody (final concentration: 4 μg/ml) (TaKaRa Bio Inc., Kusatsu, Japan) and anti-mouse osteopontin (OPN) (rabbit) antibody (final concentration: 5 μg/ml) (Immuno-Biological Laboratories Co. Ltd., Fujioka, Japan) for 60 min at RT. Cells were washed with buffer

and then incubated with goat anti-rabbit IgG FITC (1:100) (Tokyo Chemical Industry Co. Ltd., Chuo-ku, Tokyo, Japan), goat anti-rat IgG PE (1: 100) (Biomeda Corp. Ltd., San Jose, CA, USA) and Hoechst 33342 stain (DOJINDO LABORATORIES, Kamimashiki-gun, Kumamoto, Japan) for 60 min at RT in the dark. Staining was observed under a fluorescence microscope BZX710 (Keyence, Osaka, Japan).

Immunostaining showed that OPN and OC were co-localized in the control, but the expression sites of OPN and OC were not perfectly matched in HPP mice. In addition, OC was expressed uniformly throughout the cells in the control, whereas, in HPP, OC was concentrated at a single location. Moriishi et al. reported that OC was essential for orienting hydroxyapatite (HAp) crystals along collagen fibers and maintained bone strength in the long-axis direction [S4]. Heterogeneous expression of OC in HPP osteoblasts could be the cause of reduced bone strength. It is interesting to note that the decrease in tissue-nonspecific alkaline phosphatase (TNSALP) not only decreased HAp crystallinity but also affected the intracellular distribution of the bone matrix proteins that provide the bone lining, thus reducing the mechanical properties of the bone.

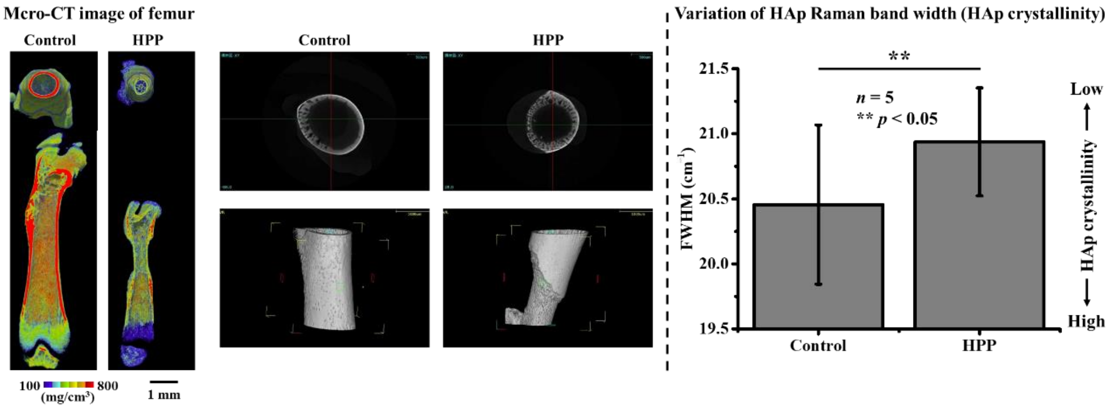

Figure S2. HPP model mice were examined using Raman and micro-CT analyses.

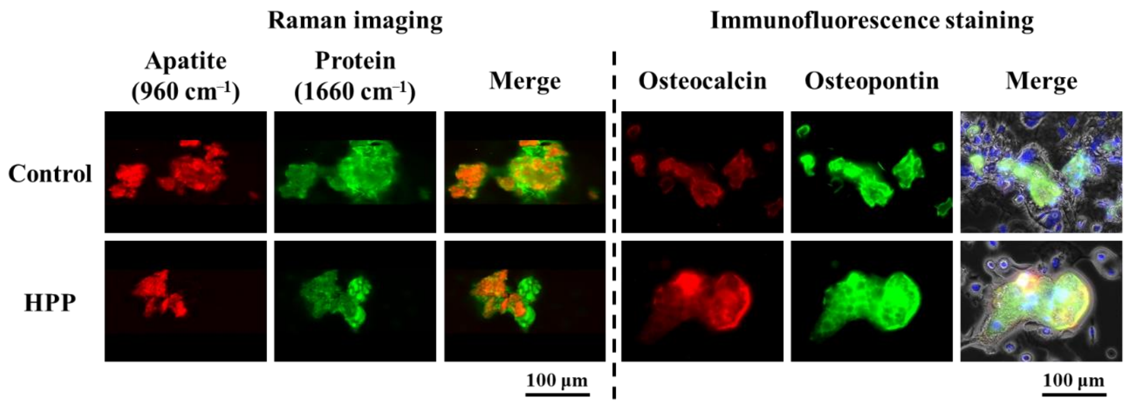

Figure S3. Fluorescence microscopic images of MSCs stained with osteopontin (green), osteocalcin (red) and Hoechst 33342 for the nuclei (blue).

References

S1. Narisawa, S.; Fröhlander, N.; Millán, J. L. Inactivation of two mouse alkaline phosphatase genes and establishment of a model of infantile hypophosphatasia. *Dev. Dyn.* **1997**, *208*(3), 432–446. DOI: 10.1002/(SICI)1097-0177(199703)208:3<432::AID-AJA13>3.0.CO;2-1

S2. Matsumoto, T.; Miyake, K.; Yamamoto, S.; Orimo, H.; Miyake, N.; Odagaki, Y.; Adachi, K.; Iijima, O.; Narisawa, S.; Millán, J. L.; Fukunaga, Y.; Shimada, T. Rescue of severe infantile hypophosphatasia mice by AAV-mediated sustained expression of soluble alkaline phosphatase. *Hum. Gene Ther.* **2011**, *22*(11), 1355–1364. DOI: 10.1089/hum.2010.210

S3. Nakamura-Takahashi, A.; Tanase, T.; Matsunaga, S.; Shintani, S.; Abe, S.; Nitahara-Kasahara, Y.; Watanabe, A.; Hirai, Y.; Okada, T.; Yamaguchi, A.; Kasahara, M. High-level expression of alkaline phosphatase by adeno-associated virus vector ameliorates pathological bone structure in a hypophosphatasia mouse model. *Calcif. Tissue Int.* **2020**, *106*(6), 665–677. DOI: 10.1007/s00223-020-00676-5

S4. Moriishi, T.; Ozasa, R.; Ishimoto, T.; Nakano, T.; Hasegawa, T.; Miyazaki, T.; Liu, W.; Fukuyama, R.; Wang, Y.; Komori, H.; Qin, X.; Amizuka, N.; Komori, T. Osteocalcin is necessary for the alignment of apatite crystallites, but not glucose metabolism, testosterone synthesis, or muscle mass. *PLOS Genet.* **2020**, *16*(5), e1008586. DOI: 10.1371/journal.pgen.1008586
